# Performance Evaluation of a Robot-Mounted Interferometer for an Industrial Environment

**DOI:** 10.3390/s20010257

**Published:** 2020-01-01

**Authors:** Istvan Biro, Peter Kinnell

**Affiliations:** Electrical and Manufacturing Engineering, Intelligent Automation Centre, Wolfson School of Mechanical, Loughborough University, Loughborough LE11 3TU, UK; i.biro@lboro.ac.uk

**Keywords:** sensor evaluation, interferometer, industrial robot arm, performance standards, robotic inspection, environmental factors

## Abstract

High value manufacturing requires production-integrated, fast, multi-sensor and multi-scale inspection. To meet this need, the robotic deployment of sensors within the factory environment is becoming increasingly popular. For microscale measurement applications, robot-mountable versions of high-resolution instruments, that are traditionally deployed in a laboratory environment, are now becoming available. However, standard methodologies for the evaluation of these instruments, particularly when mounted to a robot, have yet to be fully defined, and therefore, there is limited independent evaluation data to describe the potential performance of these systems. In this paper, a detailed evaluation approach is presented for light-weight robot mountable scanning interferometric sensors. Traditional evaluation approaches are considered and extended to account for robotic sensor deployment within industrial environments. The applicability and value of proposed evaluation is demonstrated through the comprehensive characterization of a Heliotis H6 interferometric sensors. The results indicate the performance of the sensor, in comparison to a traditional laboratory-based system, and demonstrate the limits of the sensor capability. Based-on the evaluation an effective strategy for robotic deployment of the sensor is demonstrated.

## 1. Introduction

A reliable inspection process is of key importance for high value manufacturing in order to ensure compliance with standards and consistently high-quality products. In contrast to sample-based dedicated off-line inspection, such a process is ideally production-line integrated, saving resources by eliminating the need for dedicated inspection areas and trained personnel. Additionally, this approach allows for each part to be inspected after each critical manufacturing step, detecting defects early and ensuring constant quality. Typical approaches include machine integrated sensors [1,2,3] and robot-arm-based inspection using camera vision, laser scanning or 3D pattern projection [4,5,6,7,8,9,10] (also see industrial systems such as: X4 i-Robot, Creaform Cube-R, GOM Atos Scanbox, Mertolog X4), enhanced by multi-sensor and data fusion approaches [11,12,13,14,15,16,17,18]. Allowing for fast and flexible inspection of free-form surfaces, these systems commonly lack the ability to measure sub-millimeter features with high resolution, as the sensors required for this step are typically large, heavy and require laboratory conditions and are therefore used off-line. However, recent technological advances have resulted in a new, compact generations of high resolution sensors, designed to enable greater production integration [19,20,21]. This compact nature also enables robotic deployment, as demonstrated in [22,23,24,25] through the combined use of a laser line scanner (low resolution, for large area coverage) and a scanning interferometer (Heliotis H6, high resolution, for local high resolution measurements) mounted on a robot arm. While the performance evaluation of such high resolution instrumentation in a laboratory environment has been widely addressed [26,27,28,29,30,31,32,33], the application of these sensors in non-ideal, shop-floor applications, as well as in robotic deployment, clearly demonstrates the need for re-evaluating and extending performance standards [34].

By evaluating the performance of the Heliotis H6, this paper attempts to answer three key questions: (i) how does the lightweight H6 performance compare to a lab-based coherent scanning instrument (Bruker NPFLEX); (ii) how applicable are existing performance test standards, developed for laboratory-based scanning interferometers, to a robot-mounted scenario, and what can be learned from these to improve robotic deployment; (iii) how can these standards be extended to include novel aspects arising from in-factory, robot-mounted deployment.

**Figure 1:**
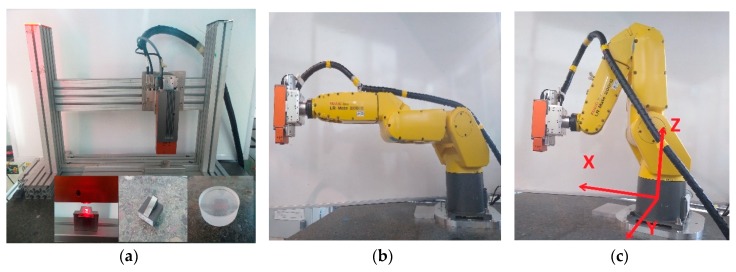
Measurement setup: Heliotis was mounted on a stable, rigid H-frame (**a**) or on the robot arm in a fully extended pose (**b**) or a compact pose (**c**). Insets on (**a**) show typical artefacts used: from right to left, an optical flat glass surface, a tilted metal flat (slip-gauge) surface and a step-artefact realized by wringing two slip-gauges of different thickness onto a third slip-gauge.

Instrument evaluation is based on noise and height profile accuracy, determined with the Heliotis H6 mounted on both a rigid stand and an industrial robot in multiple configurations (see Figure 1). The most relevant quantity (noise and data quality) to monitor is established and the most appropriate methods to measure these are highlighted. Flatness deviation, noise spread and signal strength distribution across a measured surface are characterized, for different materials, to suggest ideal feature placement within the field-of-view for best measurement results. This is extended by the evaluation of the impact of measurement speed, range and environmental factors (temperature, light) on the instrument noise level, characterizing instrument robustness in a factory environment and providing a guideline for appropriate instrument settings. Critical aspects for robotic deployment such as sample or instrument tilt and the influence of sample or robot arm vibrations are then investigated in more detail, as these factors differ most from lab-based deployments and have most impact on the quality of the obtained measurements. Based on the evaluation, a summary and strategy for optimal robotic deployment of the interferometer is provided.

## 2. Instrument Configuration and Evaluation Artefacts

### 2.1. Instruments and General Settings

The 3D interferometric surface data presented in this paper has been collected using a heliInspect^TM^ H6 Industrial White-Light Scanning Interferometer (Heliotis AG, Root, Switzerland). This instrument was manufacturer-configured to have a LINAX^®^ Lxu linear stage with 100 nm (vertical) resolution (Jenny Science AG, Rain, Switzerland) and changeable optics in form of an R5 (5 µm lateral resolution, 1.47 × 1.41 mm^2^ field of view) or an R20 (20 µm lateral resolution, 5.86 × 5.62 mm^2^ field of view) Michelson-type heliOptics^TM^ WLI6 White-Light Interferometer modules (Heliotis AG, Root, Switzerland). This interferometer has a wide range of user-settable parameters and functions to optimally support multiple measurement scenarios. In order to have comparable measurements obtained in different conditions, we have applied the standard settings (all other settable variables left at factory default values) as shown in Table 1, unless stated otherwise. These settings were chosen to use the most common and user-friendly mode of operation, and most general instrument settings, while disabling all filtering and smoothing functionality of the instrument.

The interferometer was mounted vertically (scanning motion along the vertical axis) on an H-frame built from Bosch Aluminum components and placed on a granite block for stability reasons (Figure 1a). Samples were placed horizontally under the instrument, unless stated otherwise.

For vibration evaluation, controlled vibrations were introduced using a Mini SmartShaker™ (model K2007E01, The Modal Shop, Sharonville, OH, USA). To simulate the effect of robotic deployment, the shaker was mounted on the same H-frame as the interferometer and placed under the interferometer such that the motion axis of the shaker coincided with the interferometer’s *Z* axis. A 3D printed adaptor plate was bolted to the mounting platform of the shaker. The sample was then firmly attached to this adapter plate using an adhesive. Only vibrations in the interferometer’s *Z* axis were introduced, as these are most relevant and have a complex effect on the measurements due to the combination of the interferometer’s scanning motion and the shaker vibration (along the same axis). Various angles (0, 5, 10 degrees from horizontal) of sample placement were explored using modified, appropriately angled adaptor plates, keeping the common shaker-interferometer motion axis. All vibrations generated using the shaker, driven by a signal generator, had a single-component sine-waveform with an approximate amplitude of {0, 10, 100, 500} µm and frequencies of {0, 10, 100, 500} Hz. These values were chosen to sample the typical vibration range observed in six degrees of freedom industrial robot arms. The amplitude and frequency of the applied vibrations were verified using a Polytec PDV 100 laser vibrometer (Polytec Inc., Irvine, CA, USA) and a LD1610-2 Laseroptical displacement system (Micro-Epsilon Ltd., Birkenhead, UK).

Robotic deployment was implemented using a Fanuc LR Mate 200iC robot arm with a Fanuc R30iA-Mate controller (Fanuc Ltd., Coventry, UK) and custom-written control and integration software. During robotic deployment, the interferometer was co-located with a LLT2900-50 Laser line scanner (Micro-Epsilon Ltd., Birkenhead, UK), providing surface information for accurate sensor placement (see Figure 17a).

### 2.2. Artefacts

Two different flat surfaces have been used: (i) a shiny metallic flat, in form of the highly polished surface of a calibrated slip-gauge, and (ii) an optical flat glass surface (Thorlabs PF20-03, Thorlabs Inc., Newton, NJ, USA).

Step artefacts have been created by wringing two calibrated slip-gauges with a thickness difference equal to the desired step-size side by side on top of a third (base) slip-gauge. The created step-height was verified using a Bruker NPFLEX (Bruker Corporation, Tucson, AZ, USA), equipped with a lens matching the field of view of the H6 and measuring at the same sample location as with the H6.

## 3. Evaluation and Selection of the Appropriate Performance Metrics

In order to compare instrument performance, a suitable measure had to be identified, offering valid means of instrument performance in various conditions. For this purpose, noise from a measured flat surface was used, as (i) this measure is commonly used throughout literature; (ii) it is simple to evaluate in all conditions (environmental factors, robotic or static deployment); (iii) it reflects the combined impact of all sources of noise: instrument electronic noise and noise due to external factors (e.g., vibrations); (iv) noise has been shown to be a limiting factor for the detection and evaluation of small defects [24,25]. In addition, where appropriate and possible, noise analysis was complemented by two other measures: (a) step-height measurements (using wringed slip-gauges) and (b) data quality (where good data is the ratio of data points within two times instrument Z-resolution of the fitted plane corresponding to a measured flat surface and above-instrument-electronic-noise signal strength, i.e., the ratio of measurable and non-outstanding datapoints).

Different methods of noise evaluation have been proposed throughout literature [26,30,34,35]. In an attempt to compare these methods and identify the one best suited in a robotic sensor deployment, a flat surface was measured 11 times and the noise along the instrument Z axis were evaluated using these methods: M1-3 (see below). Noise in the X and Y direction were not considered, as resolution in the XY plane is substantially lower and optics and pixel-size defined. As these methods were suggested for lab-based instruments, the Heliotis H6 was mounted on a stabile H-frame for these tests.

(M1) RMS (root mean square) method: this method estimates noise from each single measurement, by fitting a plane to the measured datapoints and calculating the noise (*Sq*(*i*)) of each measurement (i) as the root-mean-square of all point-to-plane distances (*d_pp_*, along the instrument *Z* axis): $Sq\left(i\right)=RMS\left(d_{pp}\right)$, and the overall noise (*SqR_n_*) from *n* = 11 repeated measurements as $SqR_{n}=mean\left(Sq\left(i\right)\right)\pm std\left(Sq\left(i\right)\right)$.

(M2) Subtraction method: this method uses sets of two measurements of the same surface done in quick succession to obtain topography-difference-maps by subtracting the height value of corresponding data points from the two measurements. The noise (*Sq*(*i*)) of each measurement set is then evaluated from the datapoints (*p_d_*) of the difference map as $Sq\left(i\right)=RMS\left(p_{d}\right)$ and the overall noise (*SqS_n_*) from repeating this process n times is $SqS_{n}=mean\left(Sq\left(i\right)\right)\pm std\left(Sq\left(i\right)\right)$.

(M3) Averaging method: uses *n* > 2 measurements of the same surface to calculate an averaged height map. The averaged noise of each single measurement is given as:

Sqa(i)=Sq(i)2−Sqm21−1/n

where *Sq*(*i*) and Sqm are the noise of the individual measurements and the averaged surface, respectively, calculated with the RMS method. The overall noise is then $SqA_{n}=mean\left(Sq_{a}\right)\pm std\left(Sq_{a}\right)$.

**Figure 2:**
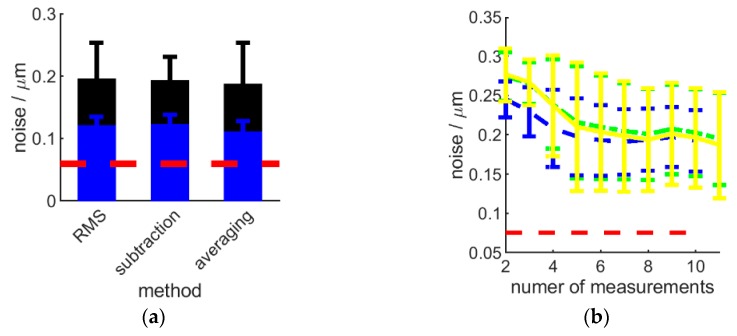
Comparison of noise estimation methods. Representative comparison of noise measures for metal (blue) and glass (black) flat surfaces are shown on (**a**) with solid bars representing the mean value and the standard deviation over 11 repeated measurements is illustrated by the single-sided positive error bar. Noise convergence for all methods (RMS: green, subtraction: blue, averaging: yellow) is shown on (**b**) for a glass surface. R5 optics and highest Z resolution used. Red dashed lines show the noise level on the averaged surface (resulting from averaging 11 single surface measurements) using the RMS method.

The noise estimates using the three methods were highly similar, as shown in Figure 2a. In addition, it was found that all three methods show convergence with increasing number of repetitions; five repeated measurements were sufficient to converge on the same noise estimate, as shown in Figure 2b. From Figure 2a, it can also be seen that the noise for glass surfaces was found to be significantly higher than in case of metal surfaces, indicating the importance of selecting a suitable test surface that is representative of intended measurement applications.

Given the similarity in performance of the three methods, to select the most suitable method, the benefits of each must be considered. For very low-noise measurements systems, where the influence of surface imperfections or flatness errors of the measurement artefact is likely to be a dominant factor for M1, it is necessary to employ either M2 or M3. M2 and M3 share a similar approach, where the influence of the measured surface is removed through averaging or subtraction of successive measurements; it is therefore expected, as observed, that they should yield similar results. However, an important factor for M2 and M3 is that the successive measurements must be made of exactly the same surface region on the measurement artefact, as they rely on the assumption that there is direct spatial correspondence between each successive measurement. This assumption is reasonable for highly stable laboratory instruments, where motion drift is limited to minor vertical movement or surface-tilt, which can be compensated by considering deviations from a least-squares plane fitted to each data set. However, if the mounting arrangement could be subject to higher levels of positional instability, including the potential for possible lateral movement, as in the case of an instrument mounted to an industrial robot arm, then M2 and M3 should not be applied. In this work, due to the need to assess performance of the instrument while mounted to a robot arm, all noise results will be calculated using method M1.

## 4. Comparing Measurement Performance with a Lab-Based Reference Instrument

In order to compare the performance of the H6 with conventional lab-based interferometric metrology equipment, noise and step-height measurements taken from the same samples were performed.

**Figure 3:**
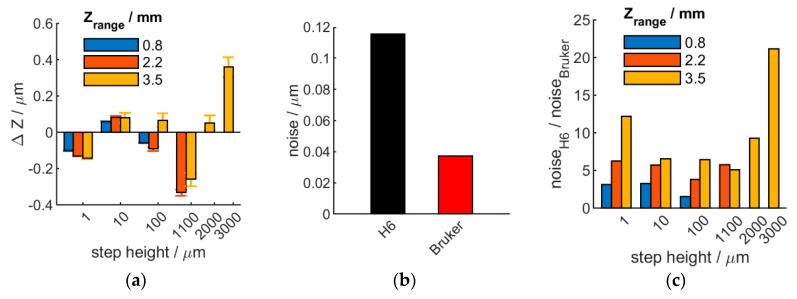
Comparative measurements of measured step-height difference (**a**), noise level, with single-sided positive error bar indicating standard deviation (**b**) and relative noise (**c**) between Heliotis H6 and Bruker NPFLEX, both evaluating the same step-height with similar field-of-view optics. Heliotis Z scan rage (in **a** and **c**): 0.83 mm (blue), 2.16 mm (red), 3.49 mm (yellow).

A series of step-heights realized by wringing two slip-gauges next to each-other, on top of a supporting slip-gauge (Figure 1a, inset) were measured using H6 and Bruker NPFLEX. The size of the step was varied through choosing slip-gauges of different thickness. The measurement was performed on the same region with both instruments and the optics was chosen to result in a similar field of view (similar sample area measured). The difference between the measured step-heights is shown on Figure 3a. The height difference for high resolution measurements (H6 Z-range of 0.8 mm) between the two instruments was found to be within H6 Z resolution of about 100 nm, throughout the measured step-height range (Figure 3a, 1–100 µm steps). Increasing Z-range on H6 allows for measuring larger step-heights but results in higher instrument-to-instrument deviations as a result of decreased instrument resolution of H6, while Bruker is able to keep high Z resolution throughout the higher Z range (Figure 3a, 1–3000 µm steps).

The measurement (Z) noise was also evaluated. H6 measurement noise was 3 times higher than the lab-based Bruker instrument, using comparable-field-of-view optics (Figure 3b). In case of larger sample height variation, the Bruker outperforms H6 in terms of noise even further. This is expected, as larger step-size artefacts force the H6 to use lower Z resolution in order to cover the necessary height range, while Bruker can maintain high Z resolution, as explained above (Figure 3c). Consequently, while the lab-based Bruker outperforms Heliotis H6 on measurement quality, the later sensor shows a comparable performance at its highest resolution and offers robotic, production-line integration options due to its low weight and substantially faster measurements (1–5 s for H6 compared to tens of seconds to minutes for Bruker, depending on artefact height). Therefore, a lab-based instrument needs to be chosen for very quality measurements, but a robot-mounted lightweight instrument presents more advantages where accuracy permits this.

## 5. Optics Choice, Sample Material and Placement within Field of View

In a robotic scenario, where initial sensor placement may suffer from robot inaccuracy, it is important to choose the right optics and account for the variation of instrument measurement capability within the field of view of the chosen optics, in order to maximize data quality. For this reason, we characterized the noise distribution, flatness deviation and signal intensity (accounting for reliability of data compared to instrument electronic noise level) across the field of view, for different optics.

### 5.1. Flatness Deviation and Noise Distribution

**Figure 4:**
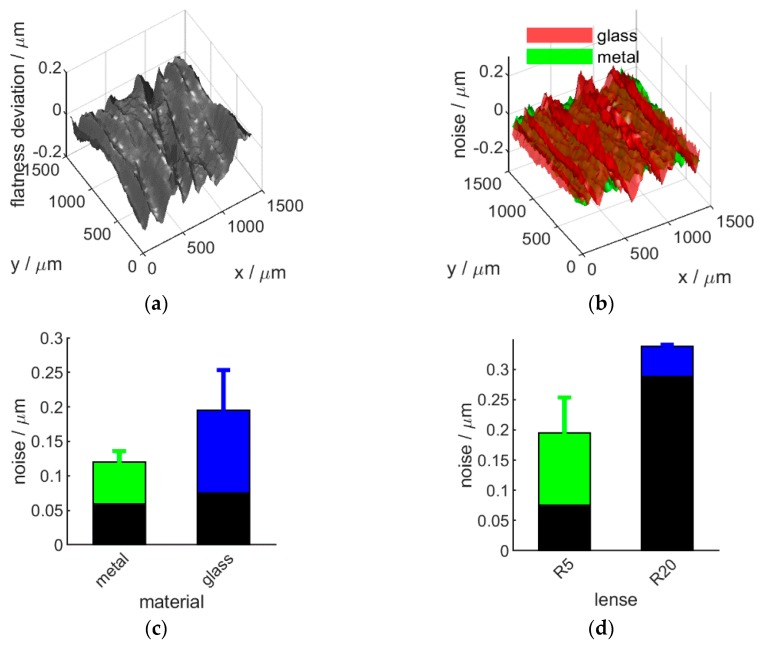
Flatness deviation (**a**), surface noise distribution (**b**) and noise level for different sample material (**c**, R5 optics) and optics (**d**, glass surface) used. Bars are the mean and the single sided positive error-bars represent the standard deviation over 11 repetitions. Black bars represent noise evaluated on the average surface (averaging the 11 single measurements).

Flatness deviation was evaluated by measuring 11 different areas of a flat glass surface, and fitting a plane on the averaged point-cloud. The point-to-fitted-plane distances represent the flatness deviation (Figure 4a). In contrast, noise distribution was evaluated by taking 11 measurements of the same flat surface area and using this data to build 10 difference-maps by subtracting subsequently measured profile data pairwise. The RMS of each corresponding point (originating from the same pixel) through these 10 difference maps was used as surface noise estimate and is shown on Figure 4b. This method is proposed as it eliminates artefact surface imperfections, similarly to noise estimation using the subtraction method.

Both flatness deviation and noise distribution are within instrument Z resolution (100 nm) and consistent throughout the field-of view, for both the high resolution R5 and the larger field-of-view R20 optics (data shown for R5 only). It is notable, however, that glass surfaces show a higher noise variation. Therefore, the overall noise level was evaluated for different materials (metal and glass) and both optics units (R5 and R20). The results (Figure 4c,d) indicate the presence of higher instrument noise for glass (compared to metal) and R20 (compared to R5) optics used.

Therefore, sensor noise and flatness deviation does not vary significantly within the field of view (Figure 4). However, the correct choice of optics can have an effect on measurement quality. Further, the influence of sample material, a commonly neglected aspect of instrument evaluation, is clearly demonstrated. This influence is further strengthened when sensor-sample orientation is not normal, as shown later (Figure 13). Neglecting this can lead to unreliable comparison of instruments and unexpected measurement results.

### 5.2. Signal Intensity

**Figure 5:**
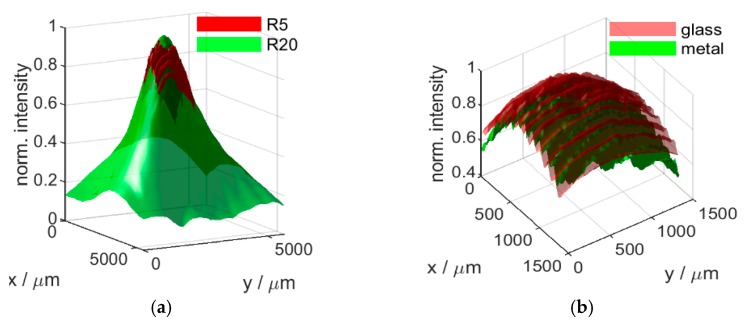
Normalized signal intensity defined as interference pattern amplitude at sample surface level for each sensor pixel for different measurements: R5 (low field-of-view) vs. R20 (high field-of-view) optics and glass surface (**a**); and glass vs. metal surface using R5 optics (**b**). Each signal intensity dataset (surface) shown is obtained by averaging 11 measurements on the same sample area, under identical conditions/settings, and is normalized using the maxima of the respective dataset. The measurements were taken using the highest instrument Z resolution.

In addition to the surface topography, the H6 also returns an amplitude (or intensity) map, characterizing signal intensity at each pixel. This is used to ensure that surface topography at the relevant pixel is obtained from a signal strength above instrument noise level (i.e., good signal-to noise ratio). Evaluated for both the fine resolution R5 and the large field-of-view R20 lens, the signal intensity was shown to strongly decay towards the edges of the field-of-view (Figure 5). Across both (R5 and R20) optics, signal intensity is observed to drop by about 20% towards the edge, within the common measured area. However, for the R20 optics, providing a wider field-of-view, this drop continues to about 70% at the edge. This observation holds for both metal and glass surfaces. In contrast to noise distribution and flatness deviation, this underlines the central placement of any feature of interest within the measured area. Additionally, features that scatter light, for example due to due to shiny or sloped surfaces, may further decrease signal intensity. These findings underline the need for an exact sensor placement by the robot, such that the feature of interest can be observed close to the middle of the field-of view.

## 6. The Effect of Mounting Strategy on the Measurement Noise

Inspection in a conventional scenario, with the H6 firmly mounted and in a robot-mounted, production-line integrated application is expected to differ in achieved performance. Therefore, we compared these two applications by evaluating measurement noise on a flat metal sample with H6 on (i) a stable H-frame, as encountered in off-line inspection and (ii) a robot arm with in different poses: fully extended and compact (Figure 1), as used in case of production-line integration. This also illustrated the impact of different mounting stability levels. Instrument settings were kept the same: R5 lens, highest resolution, 50% of max. scan speed.

**Figure 6:**
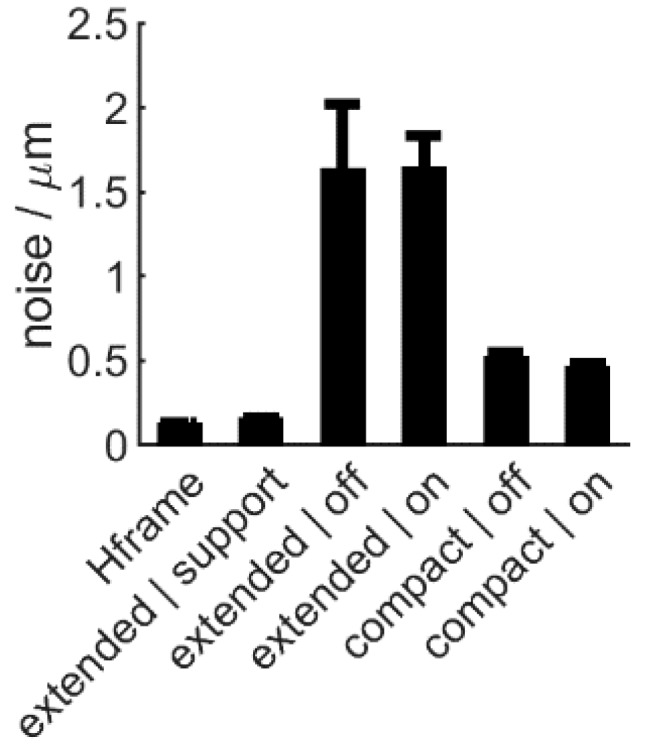
Instrument noise, with single-sided positive error bars indicating standard deviation, in various mounting scenarios: on H-frame and on the robot arm in a compact and extended pose. The arm was supported near the end-effector in one scenario to increase stability. Robot motors were on (energized) or off (unpowered).

Best performance (lowest noise) was achieved with the H6 on a rigid H-frame (Figure 6, HFame). Robot-mounted deployment resulted in a pose-dependent, decreased performance: a compact robot pose resulted in less noise then measurements performed on the fully extended arm (Figure 6). This is due to the pose-dependent vibrations of the robot arm: mechanically supporting the extended robot arm near the end-effector reduced noise significantly (Figure 6, extended/support). Notably, the state of the robot motors (on: energized or off: unpowered) did not have any significant impact on overall noise performance.

Therefore, robotic deployment is only possible at the expense of a performance decay. However, this may be acceptable if required tolerances can still be fulfilled. Using a rigid robot arm is key for retaining good performance.

## 7. The Effect of Measurement Speed and Z Range on the Measurement Noise

In addition to low weight, the high measurement speed and variable height-range are key aspects for a production-line integrated robotic deployment, where inspection speed and flexibility is of high importance. These aspects, however, can have an impact on data quality and are not considered in standard interferometer evaluation procedures. Therefore, the influence of measurement speed and scan range on instrument performance will be evaluated below, in two scenarios, with the H6 mounted on (i) a stable H-frame and (ii) a robot arm.

**Figure 7:**
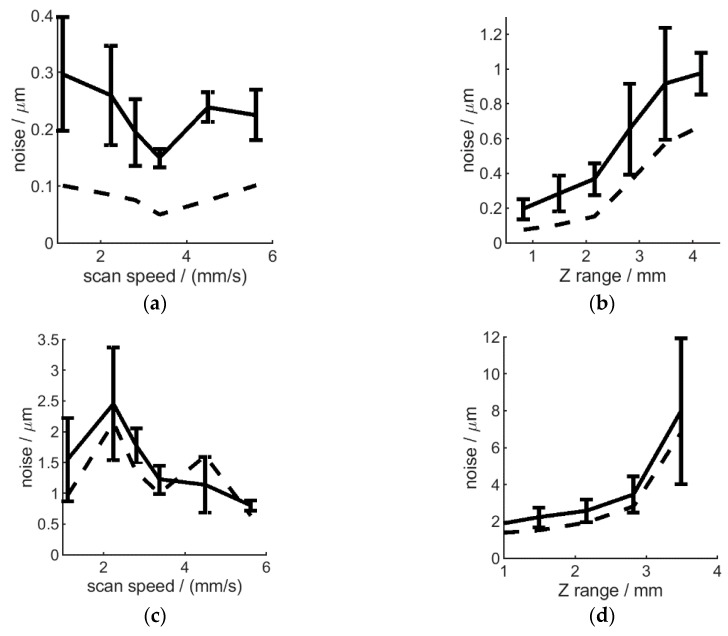
Influence of scan speed (**a**,**c**) and range (**b**,**d**) on noise level. The H6 was mounted on a stable H-frame (**a**,**b**) or on a fully extended robot arm (**c**,**d**). The mean (solid line) and standard deviation (shown by the error-bars) are shown together with the noise error of the averaged surface (dashed line). Highest Z resolution and R5 optics used.

Despite the overall difference in noise level observed for a rigid H-frame and robotic mount, instrument performance change as a function of scan speed and range was similar. The noise level was found not to be significantly altered in both scenarios, as shown in Figure 7a,c. A tendency for lower noise and less noise variation is observed for higher scan speeds, suggesting that fast robotic inspection processes are possible. This is especially applicable to surfaces with good reflectivity, allowing enough signal return to the sensor. However, fast scans may induce vibrations, as discussed later in the paper. This effect can be observed here as an increased overall noise level for robot-mounted measurements (Figure 7a) compared to H-frame based measurements (Figure 7c).

Increasing Z range allows to scan surfaces with higher height variability. However, as the instrument can only take up to 511 scans per scan, increased Z range decreases Z resolution (with the same ratio). This will also increase the noise level, as shown on Figure 7b,d. Therefore, in a robotic deployment scenario, high Z ranges should be used to locate the surface and features of interest, reposition the instrument as necessary, and the shortest possible Z range should be used for fine measurements.

## 8. Environmental Effects: Temperature and Illumination

**Figure 8:**
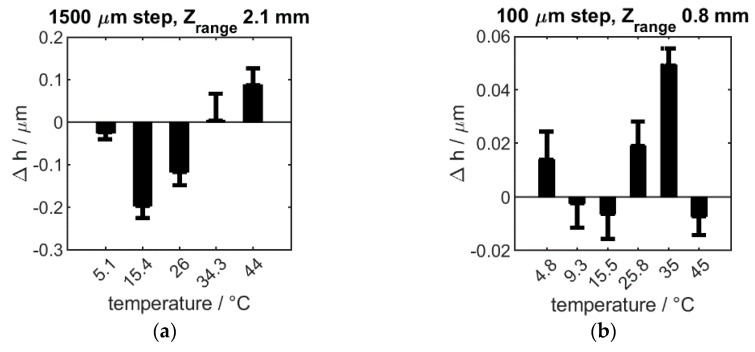
The influence of temperature on measurement quality: the difference (Δh) of the same step-height, as measured at room temperature and at the temperature shown on the x axis is plotted. This is shown for a large (**a**) and a small step size (**b**). Standard deviation is shown by the single-sided positive error bars.

Temperature stability is of high importance in in a production environment where substantial temperature changes can occur frequently. Therefore, the instrument’s performance was tested to measure the height of artefacts (wringed slip-gauges) throughout a large temperature range, results shown in see Figure 8.

The results shown in Figure 8, were collected by placing H6 on an H-frame into a test chamber. Sufficient time was allowed for the temperature to stabilize before a measurement was taken. No temperature sensitivity on relative measurements (slip-gauge difference), throughout the explored temperature range, was found. In this case, absolute height measurements are not reasonable due to thermal expansion of the entire setup. The observed thermal stability makes the instrument ideal for shop-floor deployment.

The effect of changing environmental illumination level was established using a high intensity white LED light source, introducing up to 150,000 lux of side illumination, exceeding standard room lighting by two orders of magnitude, oriented approximately 45 degrees to sample surface normal. However, no significant change in measurement noise levels was observed. This can be explained by several factors: (i) the working distance of the optical units (to sample) is relatively low (14 mm for the R5 optics and 56 mm for the R20 optics), providing a limited cover against environmental light; (ii) the LED used by the instrument in the measurement process is very bright (approximately 50,000 lux) compared to standard lighting; (iii) the instrument uses a limited bandwidth light-source centered around 650 nm, further limiting the influence of broadband environmental light and (iv) a built-in background subtraction method aids in compensating external illumination influence. Such robustness enhances production-line integrated deployment without the need for controlled lighting.

## 9. The Influence of Sensor to Sample Orientation

The advantage of robotic deployment allows for varied positioning of the interferometer. This can be used to orient the sensor adaptively relative to the local surface normal of the inspected object (ideally scanning along the surface normal). While this offers a clear advantage compared to a rigid setup, two factors need to be considered: (i) the instrument may not always be oriented to scan along the surface normal when inspecting highly curved artefacts or when the surface normal is not exactly known, leading to a sensor-sample tilt; (ii) the interferometer will not be scanning in a vertical orientation (along the gravitational field), potentially leading to conflicts with the linear stage settings. Both aspects are not traditionally included in interferometer performance evaluation and will be covered here. Instrumentation limitations in measuring sharp gradients, slopes, and artefacts with discontinuities have been previously reported [36,37] and underline the need for a detailed evaluation and inclusion in standards.

### 9.1. Sample Tilt with Vertical Sensor

**Figure 9:**
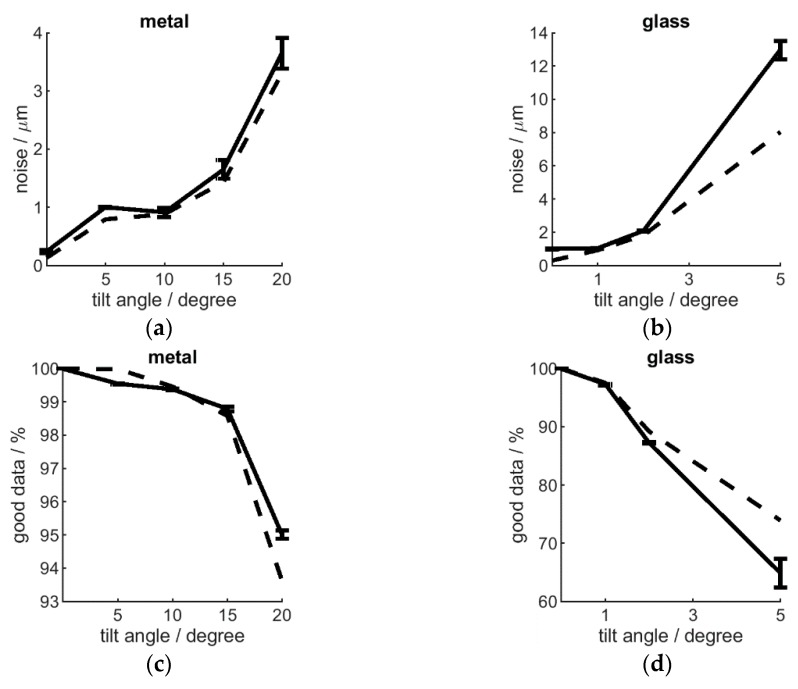
The effect of tilted sensor-to-sample orientation, showing noise (**a**,**b**) and data quality (**c**,**d**) for a metal (**a**,**c**) and a glass (**b**,**d**) surface, standard deviation is shown by the error bars. R5 optics and high Z resolution used.

Sensor-sample tilt was achieved by mounting a flat sample surface onto a support with varied tilt angle, under the H-frame-mounted (vertical) interferometer. Such a scenario resulted in a tilt angle-dependent increased noise and degraded data quality. This is especially true for glass (semitransparent) surfaces, where even few degrees of tilt result in significant loss of data. Metal surfaces can be imaged more robustly, but data quality will start decreasing substantially beyond 15 degrees of tilt (see Figure 9). Therefore, the advantage of robot-enabled articulated placement of the interferometer should be used to achieve normal-to-surface sensor positioning.

### 9.2. Tilted Sensor with Sample Normal to Sensor

**Figure 10:**
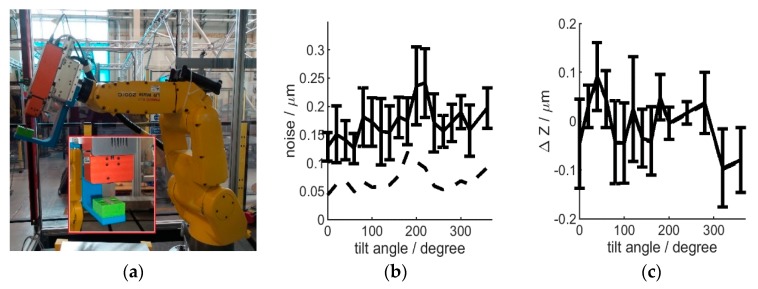
Influence of instrument tilt, setup shown in (**a**) noise results in (**b**) and step-size results in (**c**), the standard deviation is shown by the error bars.

Tilting the sensor could also influence measurements, as gravitational load on the scanning stage changes as a function of instrument orientation. To investigate this, either a flat surface or a step-artefact (formed by wringed slip-gauges, as explained previously) were mounted under the interferometer, with the surface normal permanently along the scan (*Z*) axis. The robot was used to tilt this fixed sample-sensor setup relative to the gravitational field orientation (Figure 10). Noise level extracted from the flat surface and step-size from the step-artefact were then evaluated for each tilt angle.

Surprisingly, both noise level and height profiles were shown to remain within the same range and near instrument resolution: noise was within 200 nm and Z variation was within 300 nm, with no significant correlation to tilt angle (Figure 10). This allows robotic deployment even without changes in instrument parameters (accounting for tilts) and is likely attributed to linear stage position-triggered profile acquisition during each scan, making the instrument well suited for robot-mounted operation.

## 10. Influence of Vibrations

Robotic deployment of the H6 involves exposure to non-ideal conditions, including the presence of vibrations, as illustrated earlier (Figure 6). This is actively suppressed in a laboratory environment and thus not typically accounted for during sensor evaluation.

**Figure 11:**
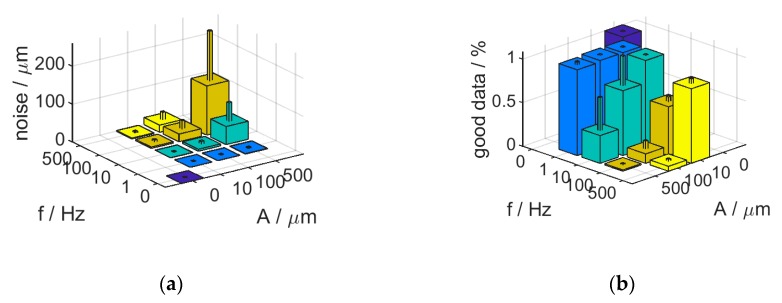
The effect of vibrating sample on noise (**a**) and data quality (**b**) for various vibration frequencies and amplitudes. The standard deviation is shown by the single-sided positive error bars.

**Figure 12:**
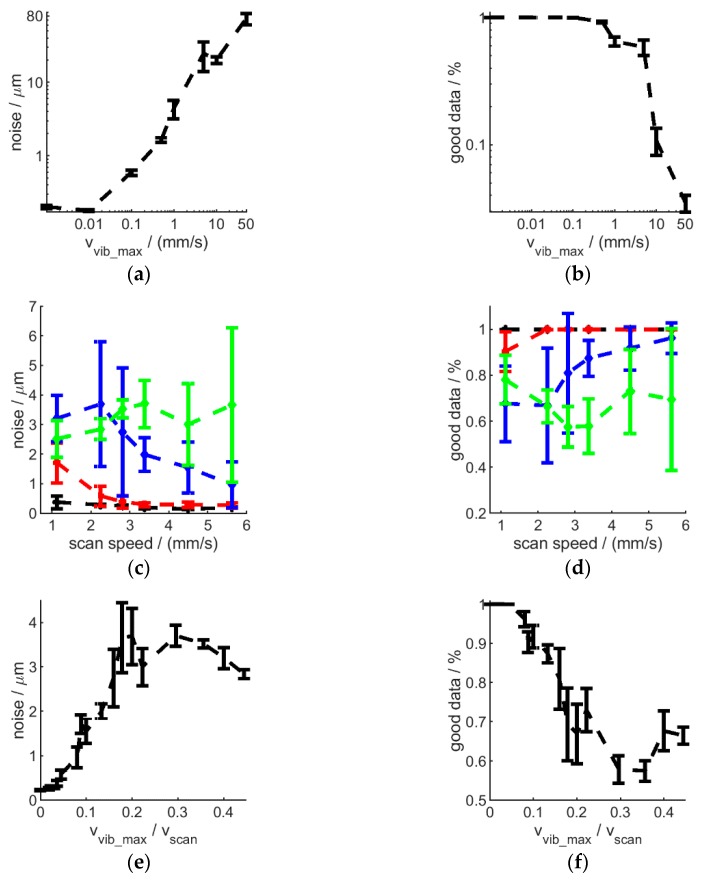
The effect of sample vibration peak velocity (**a**,**b**), measurement (scan) speed (**c**,**d**) and vibration-measurement speed ratio (**e**,**f**) on the instrument performance in terms of noise (**a**,**c**,**e**) and data quality (**b**,**d**,**f**), standard deviation is shown by the error bars. Highest instrument Z resolution and R5 lens used. Panel (**a**,**b**,**d**,**f**) vibration frequency range in {0, 1, 10, 100, 500} Hz and amplitudes in {0, 1, 10, 100, 500} µm; panel c and d: 0 Hz, 0 µm (black); 10 Hz, 10 µm (red); 45 Hz, 10 µm (blue); 100 Hz, 10 µm (green).

**Figure 13:**
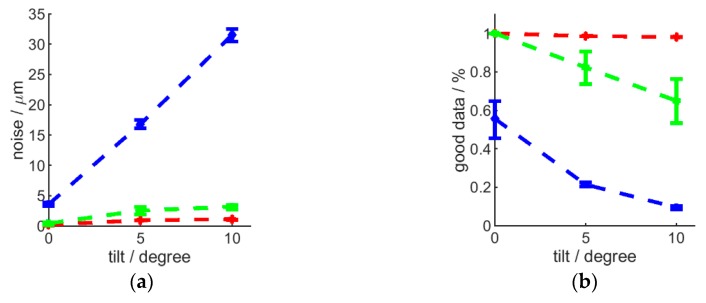
The combined effect of sample tilt and vibration on noise (**a**) and data quality (**b**); vibrations: 0 Hz, 0 µm (red); 10 Hz, 10 µm (green); 100 Hz, 10 µm (blue); the standard deviation is shown by the error bars.

In order to evaluate the effect of vibrations in more detail on surface measurements, a flat metal plane artefact was mounted on a mechanical shaker and vibrations in form of sine-waves with various amplitudes and frequencies were introduced while repeated measurements have been taken from the surface using the H6, mounted on a stable H-frame, limiting dominant vibration sources to the sample. The results are shown on (Figure 11). Measurement noise was found to increase and data quality to decrease with increasing vibration amplitude and/or frequency. While this effect is relatively small for amplitudes of up to 10 Hz, where increasing vibration frequencies only decrease measurement quality marginally, larger vibration amplitudes result in strongly degrading measurement quality with much stronger influence from vibration frequency values. In fact, noise was found to increase, and data quality to decrease, with increasing peak vibration velocity $v_{vib\_max}=A\cdot \omega$, for a harmonic vibration with a single frequency ω and amplitude A as shown on Figure 12a,b. Increasing the interferometer’s scan speed ($v_{scan}$) can be used to alleviate this (Figure 12c–f). However, for measurements to be resolved along Z within 1 µm (10 times the resolution of H6 with current settings), the scan speed needs to be at over 10 times the maximal vibration velocity. In addition, tilting the sample (or instrument) relative to the surface normal introduces further quality loss, amplified by increased vibration frequency (and amplitude) Figure 13.

## 11. Robot Arm Stability and Measurement-Induced Vibrations

In a realistic robotic scenario, multiple amplitude and frequency components need to be considered for a realistic vibration scenario, with amplitudes typically decreasing with increasing frequency, unless a specific mode is externally excited, e.g., through machining operations. Additionally, vibrations not only originate from the sample, but also from the robot arm holding the interferometer (as also shown on Figure 6).

In order to verify the relevance of such vibrations, a PDV vibrometer was used to analyze the vibrations of the 6dof robot arm in various configurations: (a) with the arm fully extended (longest horizontal state—the most susceptible to vibrations) and (b) with the arm in a compact pose and H6 close to the robot base (Figure 1). In both conditions, vibrations were measured along all three robot axis (*X*, *Y* and *Z*) but analysis was focused on vibrations along the *Z* axis as H6 was in a vertical pose (along the robot *Z* axis) and as the interferometer’s Z resolution is the highest (X and Y being pixel-defined). For each pose, vibrations were measured with (i) both the robot and H6 off, (ii) the robot motors on and H6 off, (iii) the robot motors off and H6 measuring and (iv) the robot motors on and H6 measuring, in order to identify the sources of vibrations. However, we focused on the last scenario as this represents the conditions encountered in real robotic deployment. Additionally, in order to compare the effects of a static mount and robotic application, the vibrations of the static H-frame were also measured with H6 off and measuring (Figure 1).

**Figure 14:**
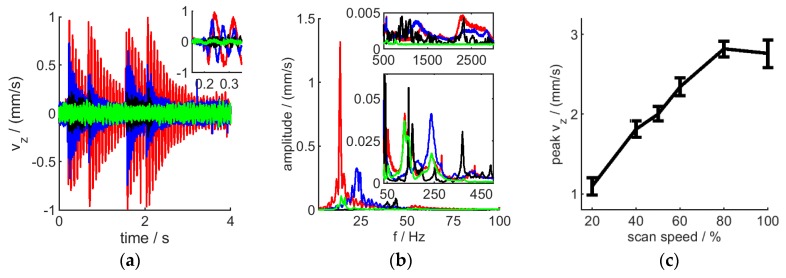
Measurement-induced vibrations. Velocity-traces (**a**) and dominant frequency components (**b**) are shown for H6 mounted on H-frame and measuring, (black); H6 inactive, on fully extended robot arm (green); H6 measuring, on fully extended robot arm (red); H6 measuring, on compact-pose robot arm (blue). Inset: zoom on first vibration cycles. (**c**): Vibration velocity peak values in function of interferometer scan speed. Error-bars represent the standard deviation of 11 repeated measurements.

Notably, vibration velocity was smallest with the robot motors and H6 off (data not shown), however, this is not a practical scenario and is disregarded. Turning on the robot motors results in excitation of the arm mostly at a small set of eigen-frequencies, mostly around 13 Hz, 130 Hz and 230 Hz (see Figure 14a,b, green curve). Operating the H6 further increases vibrations. Most prominently, starting and stopping the scan motion (two events), as well as retracting the instrument after the scan (additional two events) result in induced vibrations, decaying over time (Figure 14a, red curve). The energy transferred to the robot is mainly responsible for boosting vibrations at the eigen-frequency of the arm (Figure 14b, green and red curve), most prominently in the lower frequency domain (here at 13 Hz). In addition, higher frequency vibrations, not present without H6 activity, are also excited with less amplitude.

The frequency-components and amplitudes of such vibrations are pose-dependent: while a fully extended arm has its main vibration component around 13 Hz, this is shifted to 25 Hz with reduced amplitude in case of a more compact joint configuration (where H6 is closer to the robot base). Higher frequency components are also shifted (see Figure 14a,b, red and blue curve). Further, the scanning interferometer was found to induce significantly higher vibrations when robot-mounted, compared to deployment on a static H-frame (see Figure 14a,b, black curve), a scenario that is closer to a lab-based instrument setup. This is in line with higher overall instrument noise in a robot-mounted scenario compared to H-frame mount and depending on robot pose (see Figure 6).

In the case of a fully extended robot arm and a vmax = 2 mm/s maximal (peak-to-peak) velocity of the f = 13 Hz component, the amplitude of this vibration component can be roughly estimated to be: A = vmax/(2πf) = 25 µm. Similarly, the f = 130 Hz component with 0.07 mm/s peak velocity would have an estimated 0.1 µm amplitude. Considering the results presented above (see Section 10: Influence of vibrations), only the first component (13 Hz) is expected to reduce data quality obtained from the H6 in Z direction (but no more than by about 20%). However, the shape distortion of a measured artefact can be significant, considering the manufacturer-provided instrument resolution of 0.1 µm along the *Z* axis and 5 µm in the X and Y dimensions (in case of the high resolution R5 optics). In comparison, robot vibrations without H6 actively measuring are negligible. Thus, robot arm stiffness and pose are critical to reducing vibrational effects on robot-mounted scanning interferometers.

Scan speed was found to increase peak vibration velocity (Figure 14c), however, increased scan speed was still found to reduce overall measurement noise (see Figure 7) even with H6 on a fully extended robot arm, as long as the instrument scan axis is placed along the surface normal, allowing for a fast capture of the entire surface (i.e., capturing all surface points within the same small fraction of the total scan distance and therefore being less sensitive to vibration caused displacement).

Induced vibrations could be reduced by increasing the acceleration and deceleration distance of the scanning interferometer’s stage or by decreasing scan velocity. However, both measures will lead to an increased measurement time. Alternatively, active vibration damping can be considered. This is not trivial, as induced vibrations strongly depend on the settings of the interferometer and the robot pose (altering both susceptibility and vibration characteristics). Software-based compensation could, therefore, be the preferred path. This could be done by measuring the vibrations with a sensor (e.g., accelerometer) and re-calculating (correcting) the *Z* values assigned to each image taken by the interferometer during its scan motion before surface data is reconstructed. Alternatively, the effectiveness of vibration-removal strategies originally developed for laboratory-based instruments can be tested [33,38,39,40].

**Figure 15:**
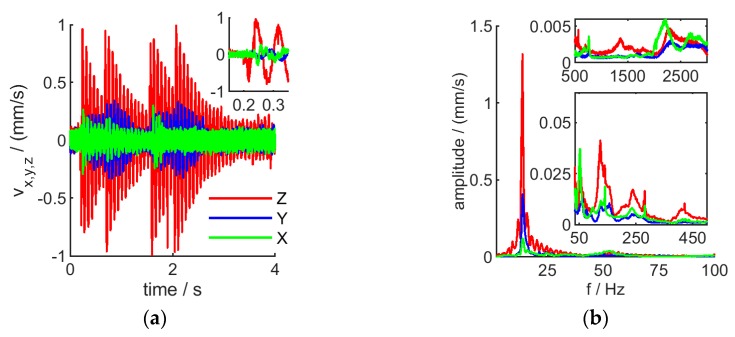
Velocity-traces (**a**) and dominant frequency components (**b**) of vibrations along the *X*, *Y* and *Z* axis, respectively, shown for H6 mounted on a fully extended robot arm.

It should also be noted that the interferometer was set up to scan along the robot’s *Z* axis during our experiments. This clearly led to strong vibrations along the *Z* axis, compared to the other axis (Figure 15). However, with the H6 tilted (for e.g., in order to measure a curved object), X and Y vibrations can exceed those along Z. Therefore, any data interpretation or compensation attempt must also include the sensor pose.

## 12. Summary and Robotic Deployment Strategy

In traditional laboratory-based instruments, environmental factors such as ambient light and temperature are well controlled. In addition, the orientation of instruments is carefully constrained on specially designed tables and mounting frames, often with active vibration isolation. In the case of a robot-mounted instrument, subject to an industrial environment with variable factory conditions, the impact of these factors must also be considered when evaluating performance.

Results in this paper have shown that the Heliotis H6 instrument was reasonably robust to key environmental factors (light and temperature). In addition, it was also found to be not adversely affected by non-vertical deployment. However, instrument noise in a robot-mounted scenario was shown to be higher than in the case of rigid instrument mounting, due to arm vibrations (natural vibrations and vibrations induced by the scanning interferometer during a scan motion). However, it was shown that this effect can be reduced by using a compact robot pose that keeps the interferometer close to the robot base; by extension using a robot with a greater level of structural rigidity is also likely to provide similar benefits although more work is needed to demonstrate this. Increasing scan speed and using the lowest possible measurement range was also shown to be beneficial to reducing the impact of vibrations and increasing measurement quality.

In addition to environmental factors, it was also shown that positioning the interferometer with its scan axis parallel to the surface normal is of key importance in order to avoid data loss (especially in case of certain materials such as glass) and reduce the effects of the robot arm vibrations. It is suggested that this placement can be realized using an additional, lower resolution sensor, such as a laser line scanner, to provide a first surface localization and shape estimate. Such a sensor can also be used to obtain fast surface data allowing for the detection of potential features of interest [24].

**Figure 16:**
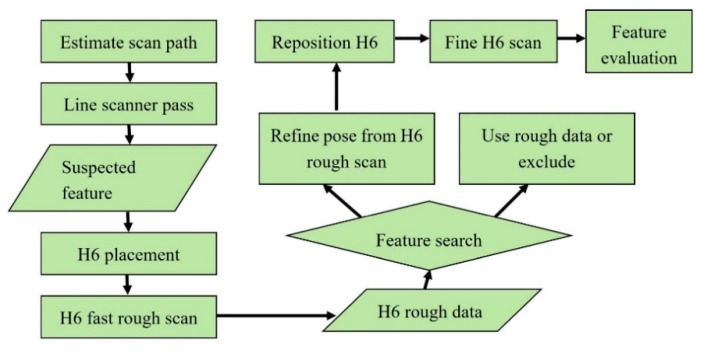
Proposed robotic inspection strategy.

To illustrate how the performance evaluation data can be used to improve a measurement or inspection process, the findings of the evaluation completed here were used to propose an improved robotic deployment strategy that is illustrated using the flow diagram shown in Figure 16. The process starts by using surface data acquired by an additional sensor, such as the suggested laser line scanner, which can be used to place the interferometer over the identified feature with fair orientation; considering distance to the surface, normality to the surface and location of the feature of interest within the measurement field of view. Following this, a first low-resolution, increased-range, high-speed interferometer scan can be used to obtain more accurate surface topography and localize the feature of interest in the interferometer’s field of view. Based on this information, the robot arm can be used to correct the interferometer pose, allowing for fine positioning, that ensures the interferometer is normal to the target surface with the feature of interest centered in the field-of-view. As signal intensity was found to be highest in this region, such a feature placement allows for obtaining optimal signal-to-noise ratio at the region of interest, increasing measurement quality and subsequent feature evaluation.

**Figure 17:**
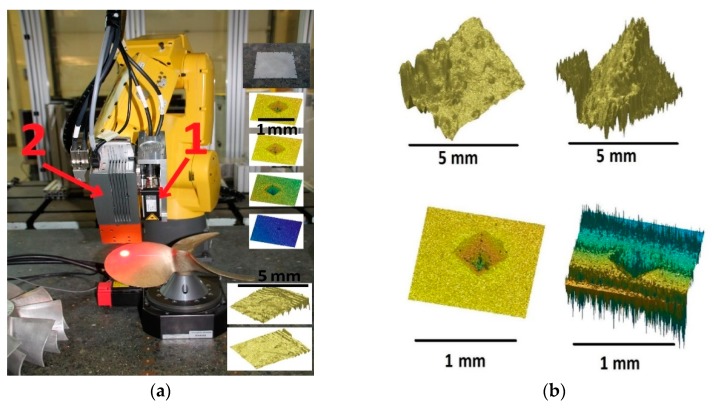
Robotic deployment demonstrator set-up (**a**) and measurement samples taken using the H6 (a-insets, **b**) from various objects: a metal plate with various-size diamond-shape indents and a boat propeller. On (**a**), 1 marks the laser line scanner and 2 marks the H6 interferometer. The right figure shows examples of good quality scans (left column) and bad quality data due to improper instrument alignment (right column).

To demonstrate the feasibility and effectiveness of the above strategy, the H6 was co-mounted onto a robot arm with a laser line scanner as shown in Figure 17. Two different artefacts, the surface of a marine propeller and diamond indentations on a flat aluminum plate, were measured using the laser line scanner, and features of interest were identified and inspected in detail using the interferometer. This was completed using the described multi-stage strategy described above (Figure 16). Sample results are shown on Figure 17b, in the left-hand side images, which show sub-millimeter features could be measured with high resolution and good data quality was achieved using the suggested strategy. Deviating from this strategy resulted in poor data quality as shown in Figure 17b, in the right-hand images, where the same features were measured, but the measurements suffer from significant noise and other erroneous measurement features.

## 13. Conclusions

This work has presented a detailed evaluation of a commercially available lightweight interferometric sensor that has been integrated with an industrial robot. The sensor performance was comprehensively studied and compared to a high-performance laboratory-based coherent scanning instrument (Bruker NPFLEX). As current evaluation approaches are generally limited to laboratory-based systems, conventional approaches and metrics for the evaluation of laboratory-based scanning interferometers were considered for their suitability when characterizing robot-mounted instruments. In addition, it was found to be necessary to extend considerably the scope of the evaluation in order to consider the wide range of potentially adverse environmental effects resulting from robotic in-factory deployment.

The comprehensive evaluation that was undertaken has allowed the performance envelope of the sensor (Heliotis H6), when used in conjunction with an industrial robot, to be well characterized. While the performance of the robot mounted system is lower than the laboratory based alternative, it was shown that with the knowledge gained from a detailed evaluation, suitable applications for the robot-mounted systems can be defined. It was also demonstrated that the pose of the robot, and placement of the sensor relative to the target surface are critical for achieving optimal measurement results; illustrating the need for comprehensive measurement system evaluations when defining the performance of robot-mounted high precision measurement systems.

Finally, the work has demonstrated that through a full performance evaluation of high-resolution robot-mounted sensors, it is possible to define effective deployment strategies that minimize sensitivity to adverse environmental, or robot-induced factors that would otherwise severely limit performance and the applicability of the instrument or sensor.

## Figures and Tables

**Table 1:** H6 instrument settings used. All other settings left at factory default.

Parameter Name, Value	Reason/Effect/Explanation
CamMode = 7 (alternative 5)	Camera mode 7, the most intuitive and general setting, returning (3-point interpolated) 3D height map (surface topography) of the measured sample, together with the corresponding signal amplitude (intensity) map. Camera Mode 5 returns the (non-interpolated) height and amplitude map at the surface level and the same data measured at heights close to (under and above) the detected sample surface level (for each sensor pixel). Default value was 7.
SensTqp = 982	This variable allows control over the vertical scanning (motion) speed. A value of 982 represents 2.81 mm/s (chosen as 50% of maximal speed in highest resolution mode: SensNavM2 = 1).
SensNavM2 = 1 (alternatives 3, 5, 7, 9)	This defines the Z scan range (max. sample height). A value of 1 means 0.83 mm Z range and represents the highest Z resolution. A value of 9 represents a Z scan range of 3.49 mm. Default value used was 1.
SensNFrames = 511	Number of Z levels measured in the selected Z range (see SensNavM2). 511 is the maximum allowed.
SensDeltaExp = var	Sensor exposure level. This is custom-set in each measurement condition, in order to achieve a peak signal amplitude of ~400 (strong signal-to-noise ratio, but avoids saturation).
BSEnable = 1	Background subtraction feature enabled.
MinEnergWin = 0	Disables an advanced built-in smoothing filter in CamMode 7.
ExSimpMaxHwin = 10	The number of measurements returned around an amplitude peak (representing sample surface level at the sensor pixel in question). Used only in Cam Mode 5.
DdsGain = 2	Default gain.
FWHMnFrame = 0	Disables data filtering at instrument pre-processing stage.

## References

[1] Gao W., Haitjema H., Fang F.Z., Leach R.K., Cheung C.F., Savio E., Linares J.M. (2019). On-machine and in-process surface metrology for precision manufacturing. CIRP Ann..

[2] Takaya Y. (2013). In-process and on-machine measurement of machining accuracy for process and product quality management: A review. Int. J. Autom. Technol..

[3] Pilný L., Bissacco G., De Chiffre L. Validation of in-line surface characterization by light scattering in Robot Assisted Polishing. Proceedings of the 3rd International Conference on Virtual Machining Process technology (VMPT).

[4] Chen W., Xiong W., Cheng J., Gu Y., Li Y. Joint texture and geometry analysis for robotic adaptive visual inspection. Proceedings of the TENCON IEEE Region 10 International Conference.

[5] Kiraci E., Franciosa P., Turley G.A., Olifent A., Attridge A., Williams M.A. (2017). Moving towards in-line metrology: Evaluation of a Laser Radar system for in-line dimensional inspection for automotive assembly systems. Int. J. Adv. Manuf. Technol..

[6] Montironi M., Castellini P., Stroppa L., Paone N. (2014). Adaptive autonomous positioning of a robot vision system: Application to quality control on production lines. Robot. Comput. Manuf..

[7] Diao S., Chen X., Luo J. (2018). Development and Experimental Evaluation of a 3D Vision System for Grinding Robot. Sensors.

[8] Wagner M., Hess P., Reitelshoefer S., Franke J. 3D Scanning of Workpieces with Cooperative Industrial Robot Arms. Proceedings of the ISR 2016: 47st International Symposium on Robotics.

[9] Borangiu T., Dumitrache A. (2010). Robot Arms with 3D Vision Capabilities. Advances in Robot Manipulators.

[10] Yin S., Ren Y., Guo Y., Zhu J., Yang S., Ye S. (2014). Development and calibration of an integrated 3D scanning system for high-accuracy large-scale metrology. Measurement.

[11] Hackett J.K., Shah M. Multi-sensor fusion: A perspective. Proceedings of the IEEE International Conference on Robotics and Automation.

[12] Esteban J., Starr A., Willetts R., Hannah P., Bryanston-Cross P. (2005). A Review of data fusion models and architectures: Towards engineering guidelines. Neural Comput. Appl..

[13] Huang Y.-B., Lan Y.-B., Hoffmann W.C., Lacey R.E. (2007). Multisensor data fusion for high quality data analysis and processing in measurement and instrumentation. J. Bionic Eng..

[14] Weckenmann A., Jiang X., Sommer K.-D., Neuschaefer-Rube U., Seewig J., Shaw L., Estler T. (2009). Multisensor data fusion in dimensional metrology. CIRP Ann..

[15] Wagner M., Heß P., Reitelshofer S., Franke J. Data Fusion Between a 2D Laser Profile Sensor and a Camera. Proceedings of the 12th International Conference on Informatics in Control, Automation and Robotics.

[16] Abidi M., Eason R., Gonzalez R. (1991). Autonomous robotic inspection and manipulation using multisensor feedback. Computer.

[17] Sahu O.P., Biswal B.B., Mukherjee S., Jha P. (2014). Multiple Sensor Integrated Robotic End-effectors for Assembly. Procedia Technol..

[18] Gronle M., Osten W. (2016). View and sensor planning for multi-sensor surface inspection. Surf. Topogr. Metrol. Prop..

[19] Danzl R., Lankmair T., Calvez A., Helmi F. Robot solutions for automated 3D surface measurement in production. Proceedings of the 18th International Congress of Metrology.

[20] Heliotis A.G. (2006). New Swiss spin-off company unveils high resolution tomographic and topographic imaging system. Sens. Rev..

[21] Lambelet P. (2011). Parallel optical coherence tomography (pOCT) for industrial 3D inspection. Opt. Meas. Syst. Ind. Insp. VII.

[22] Domaschke T., Schüppstuhl T., Otto M. Robot guided white light interferometry for crack inspection on airplane engine components. Proceedings of the International Conference ISR and 45th International Symposium on Robotics ISR/ROBOTIK.

[23] Biro I., Sharifzadeh S., Tailor M., Kinnell P., Jackson M. The evaluation of a multi-sensor robotic visual inspection system. Proceedings of the 16th International Conference of the European Society for Precision Engineering and Nanotechnology, EUSPEN.

[24] Sharifzadeh S., Biro I., Lohse N., Kinnell P. (2018). Abnormality detection strategies for surface inspection using robot mounted laser scanners. Mechatronics.

[25] Sharifzadeh S., Biro I., Lohse N., Kinnell P. Robust Surface Abnormality Detection for a Robotic Inspection System. Proceedings of the 7th IFAC Symposium on Mechatronic Systems.

[26] Giusca C.L., Leach R., Helary F., Gutauskas T., Nimishakavi L. (2012). Calibration of the scales of areal surface topography-measuring instruments: Part 1. Measurement noise and residual flatness. Meas. Sci. Technol..

[27] Giusca C.L., Leach R., Helery F. (2012). Calibration of the scales of areal surface topography measuring instruments: Part 2. Amplification, linearity and squareness. Meas. Sci. Technol..

[28] Giusca C.L., Leach R. (2013). Calibration of the scales of areal surface topography measuring instruments: Part 3. Resolution. Meas. Sci. Technol..

[29] Leach R., Giusca C., Rickens K., Riemer O., Rubert P. (2014). Development of material measures for performance verifying surface topography measuring instruments. Surf. Topogr. Metrol. Prop..

[30] Giusca C.L., Leach R.K. (2013). Calibration of the metrological characteristics of Coherence Scanning Interferometers (CSI) and Phase Shifting Interferometers (PSI). Measurement Good Practice Guide 127.

[31] Leach R., Giusca C.L. (2012). Determination of the metrological characteristics of optical surface topography measuring instruments. Opt. Micro Nanometrol. IV.

[32] Kiselev I., Kiselev E.I., Drexel M., Hauptmannl M. (2018). Precision of evaluation methods in white light interferometry. Correlogram correlation method. Measurement.

[33] Troutman J., Evans C.J., Ganguly V., Schmitz T.L. (2014). Performance evaluation of a vibration desensitized scanning white light interferometer. Surf. Topogr. Metrol. Prop..

[34] Barker A., Syam W.P., Leach R.K. Measurement noise of a coherence scanning interferometer in an industrial environment. Proceedings of the ASPE 2016 Annual Meeting.

[35] Maculotti G., Feng X., Galetto M., Leach R. (2018). Noise evaluation of a point autofocus surface topography measuring instrument. Meas. Sci. Technol..

[36] Gao F., Leach R.K., Petzing J., Coupland J.M. (2008). Surface measurement errors using commercial scanning white light interferometers. Meas. Sci. Technol..

[37] Coupland J., Leach R., Su R., Wang Y. (2017). On tilt and curvature dependent errors and the calibration of coherence scanning interferometry. Opt. Express.

[38] Mun J.I., Jo T., Kim T., Pahk H.J. (2015). Residual vibration reduction of white-light scanning interferometry by input shaping. Opt. Express.

[39] Liu Q., Yue X., Li L., Zhang H., He J. (2018). Robust phase-shifting interferometry resistant to multiple disturbances. J. Opt..

[40] Park J., Kim S.-W. (2010). Vibration-desensitized interferometer by continuous phase shifting with high-speed fringe capturing. Opt. Lett..

